# Circular Anterior Lens Capsule Rupture Caused by Blunt Ocular Trauma

**DOI:** 10.4103/0974-9233.61227

**Published:** 2010

**Authors:** Habib Dezhagah

**Affiliations:** Department of Ophthalmology, Apadana Hospital, Ahwaz, Iran

**Keywords:** Anterior Capsule Rupture, Ocular Trauma, Traumatic Cataract

## Abstract

A 16 year old male experienced blunt ocular trauma causing rupture of the anterior lens capsule and mature cataract development. The trauma was due to a stone that impacted the left eye. In an otherwise clear lens, an anterior lens capsule defect formed post-trauma that progressed to a mature cataract over four months reducing distance vision from 20/125 to hand motion. The patient underwent phacoemulsification with posterior chamber intraocular lens implantation in the left eye. One year postoperatively, the vision in the left eye increased to 20/25 without correction. This is a rare case of cataract formation due to a defect in the anterior lens capsule caused by blunt ocular trauma.

## INTRODUCTION

Blunt ocular trauma frequently leads to damage of the cornea, lens and retina. However there is relative paucity of literature on isolated anterior lens capsule rupture following blunt ocular trauma. For example, there are only two reports describing four patients with isolated anterior lens capsule rupture due to blunt ocular trauma in the peer reviewed literature.^1^,^2^ The current case report presents a patient who developed a circular defect in the anterior lens capsule after blunt trauma to his left eye that resulted in cataract formation.

## CASE REPORT

A 16-year-old male experienced blunt trauma to his left eye due to a stone. Six hours after the trauma he presented to the emergency department complaining of ocular pain. At presentation the best corrected visual acuity (BCVA) was 20/20 in the right eye and 20/125 in the left eye. The right eye was normal, however the left eye had mild lid edema, 2+ conjunctival injection, a 1 mm by 2 mm corneal abrasion located peripherally at the 6 o'clock meridian and 4+ red blood cells in anterior chamber.

**Figure 1:**
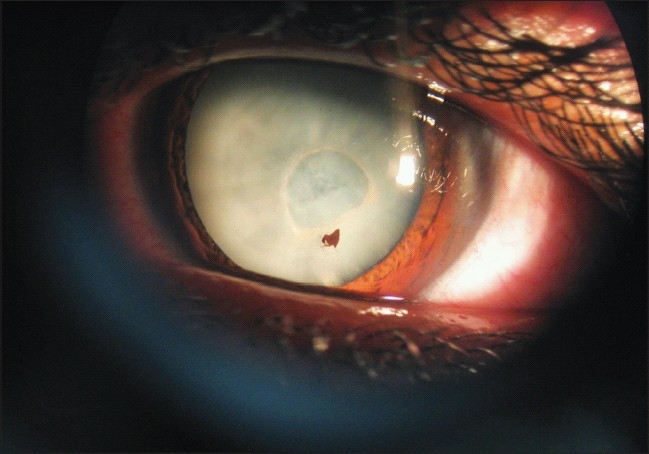
Mature cataract that developed 4 months after trauma. Note the iris pigment on the lens and relatively circular defect on the anterior lens capsule

Pupillary dilation revealed iris pigment on the anterior lens capsule (ALC), and a 3 mm diameter paracentral circular shaped defect located inferotemporally on the ALC [Figure 1]. The posterior capsule was intact and the lens cortex and nucleus were mostly clear. The remaining examination including dilated funduscopy and computed tomographies of the orbits was normal.

The patient refused to undergo lens removal with posterior chamber intraocular lens (PCIOL) implantation. Four weeks after the trauma visual acuity decreased to count fingers at 6 meters in the left eye. Subsequently the patient was lost to follow up for approximately 3 months. Four months after the trauma, he was referred to our center with a mature cataract and reduction of vision to hand motion in the left eye [Figure 1].

The patient agreed to undergo surgery after the initial consultation. Intraoperatively, a radial incision was created within the anterior capsule defect and a round, 5 mm capsulorrhexis was performed followed by phacoemulsification and implantation of a foldable PCIOL in the capsular bag.

**Figure 2:**
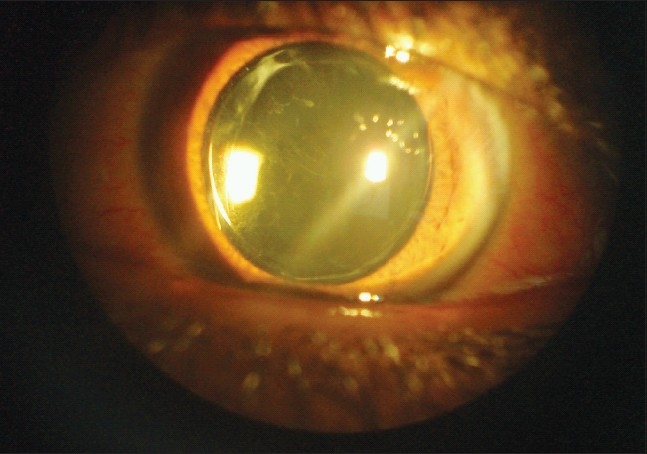
One year postoperative. The lens is well-centered in the bag. Note the trace posterior capsular opacity.

One year postoperatively, the BCVA in the right eye remained 20/20 and improved in the left eye to 20/25 with a manifest refraction of right eye was +0.25-0.25x90° and refracton of left eye is +0.25 – 0.75 × 85°. The IOL was in the bag and well centered, however trace posterior capsular opacity was present on slit lamp examination [Figure 2].

## DISCUSSION

Damage to the crystalline lens by blunt ocular trauma can result in lens dislocation, lens subluxation, lens opacity and posterior capsule rupture.^3^–^6^ However only two reports of isolated anterior lens capsule rupture due to blunt ocular trauma have been published to date.^1^,^2^ Zabriskie *et al*.^1^ reported a 10-year-old female with anterior lens capsule rupture after automobile airbag trauma. Banitt *et al*.^2^ reported 3 cases of blunt trauma causing rupture of the anterior lens capsule with subsequent cataract formation.

By comparison, posterior lens capsule damage due to blunt trauma has been reported relatively frequently.^3^–^7^ Lee *et al*. reported a case of isolated posterior capsule r upture and traumatic cataract caused by blunt ocular trauma.^5^ Yasukawa *et al*. reported a case of r uptured posterior capsule due to nonpenetrating ocular injury.^6^ Gampanella *et al*.^7^ reported two cases of traumatic cataract that developed posterior capsule r upture. None of these cases were due to penetrating or perforating injuries to the globe. Gampanella *et al*.^7^ proposed a mechanism for the posterior capsule rupture due to blunt trauma based on the anatomical relationships of the vitreo-lenticular interface. Their theory is based on the obser vation that Wiegert's ligament, an attachment of the anterior cortical vitreous to the posterior lens capsule, is most adherent in the midperipheral region of the lens of the young and loses its strength with ageing. They propose that rapid compression and expansion forces directed along the anteroposterior axis of the eye may avulse the central region of the posterior lens capsule.^7^

An explanation of anterior lens rupture has been proposed by Bannitt *et al*. who hypothesize that the anterior lens capsule may be torn due to direct contusion from rapid focal indentation of the cornea onto the lens (coup injury) or due to a rapid anteriorly directed rebound of vitreous due to fluid-mechanical forces bursting open the anterior capsule (contrecoup injury).

In the current case, the corneal abrasion was located almost at the corneo-limbal junction at the 6 o'clock meridian. The location of this abrasion was likely due to reflex eyelid closure and Bell's phenomenon just prior to the trauma which left this part of the cornea uncovered. Although the location of the corneal abrasion was not coincident with the ALC defect, direct contusion from rapid focal indentation of the cornea and tearing of the ALC proposed by Banitt *et al*.^2^ explains this observation.

In the current case, the vision in the left eye was initially preserved to some degree (20/125) because the lens remained relatively clear despite the anterior capsular defect. The defect in the capsule, caused gradual lens hydration resulting in mature cataract formation with loss of vision to hand motion by 4 months after the initial insult.

Blunt ocular trauma can cause isolated anterior capsule damage and subsequent cataract formation due to lens hydration. The first step to appropriate management is the recognition of this rarely reported occurrence.
